# The (Future) Role of Self‐Help Interventions in the Treatment of Eating Disorders: Commentary on Linardon et al. (2025)

**DOI:** 10.1002/eat.24470

**Published:** 2025-05-19

**Authors:** Steffen Hartmann

**Affiliations:** ^1^ Department of Psychology Heidelberg University Heidelberg Germany

**Keywords:** eating disorders, ecological momentary assessment, mechanisms of change, self‐help interventions, transdiagnostic treatments, web‐based treatments

## Abstract

Eating disorders are associated with a high burden, functional impairment, and many comorbidities. Thus, evaluating the effectiveness of self‐help interventions solely based on eating disorder symptom reduction limits their practical impact. The recent meta‐analysis by Linardon and colleagues investigated the effectiveness of self‐help interventions on general mental health outcomes. Although the results were promising, they also underscore several challenges encountered in research on self‐help interventions for eating disorders. These include small effect sizes, limited data on clinically diagnosed samples, and insufficient evidence regarding mechanisms of change. In the future, adopting a transdiagnostic, process‐oriented perspective and taking advantage of the flexibility of self‐help interventions may lead to more effective treatments. Ultimately, this could help generalize treatment effects across various mental health symptoms and enhance overall quality of life in patients with eating disorders.

Eating disorders are associated with severe mental health symptoms and considerable functional impairments; still, they often remain untreated, increasing their overall burden. Self‐help interventions may help close this treatment gap by complementing established psychotherapy and addressing some limitations, such as accessibility and scalability. However, although this potential has been long acknowledged, robust empirical evidence for their effectiveness, including meta‐analyses, has only recently emerged. Adding to previous meta‐analyses demonstrating the benefits of self‐help interventions in reducing eating disorder symptoms, the recent meta‐analysis by Linardon, Jarman, et al. (2025) examined the effects of self‐help interventions on general mental health outcomes, including quality of life, distress, and comorbid symptoms.

In summary, their findings indicate that in pre‐selected samples of individuals experiencing eating disorder symptoms, access to a self‐help intervention reduced depressive and anxiety symptoms, distress, and low self‐esteem. However, it did not improve quality of life or clinical impairment. In clinical samples of patients diagnosed with a full‐threshold eating disorder, small but significant effects were observed for distress, clinical impairment, and quality of life. For depression, anxiety, or self‐esteem, no significant effects were found (Linardon, Jarman, et al. 2025). These findings underline the potential of self‐help in eating disorder treatment. As these interventions are easily scalable, they can potentially reach a large number of participants. Hence, small statistical effects may lead to considerable practical effects and, as discussed, self‐help interventions, especially when widely disseminated online, may have a significant public health impact.

## 1 Challenges in Research on Self‐Help Interventions for Eating Disorders

At the same time, the meta‐analysis by Linardon, Jarman, et al. (2025) also highlights the challenges of self‐help for eating disorders: The observed effects were relatively small and not consistently found for all included outcomes, raising concerns about whether these modest improvements at the group level translate into practically meaningful changes in individuals' everyday lives. Therefore, despite these promising results, a greater emphasis on the effects of self‐help interventions beyond mere symptom reduction is needed (Linardon, Jarman, et al. 2025). To date, research on self‐help interventions has primarily aimed to evaluate their impact on eating disorder symptoms, resulting in less evidence regarding general mental health outcomes. Accordingly, in another recent meta‐analysis on the effectiveness of self‐help interventions in reducing eating disorder outcomes, the authors could include a substantially larger number of studies, thus yielding more robust results (Linardon, Liu, et al. 2025). Critically, focusing on eating disorder symptom reduction does not reflect the experiences of many individuals affected, who often face a decline in quality of life, considerable impairments in everyday functioning, and comorbid symptoms. For them, improving overall mental well‐being might be just as important as alleviating disorder‐specific symptoms, and treatment programs not addressing generalized symptom patterns might be limited in terms of (long‐term) acceptability, effectiveness, and remission rates.

Relatedly, the current meta‐analysis underlines the limited evidence on the effectiveness of self‐help for clinically diagnosed eating disorders (Linardon, Jarman, et al. 2025). The authors distinguished between samples without a formal diagnosis (pre‐selected) and samples formally diagnosed (clinical). This distinction allows for estimating separate effect sizes for the two populations. However, as many of the primary studies in the pre‐selected samples did not include a formal diagnosis procedure, whether and how many participants fulfilled the formal criteria for an eating disorder diagnosis cannot be determined. Thus, evaluating these distinctive effects is challenging. Moreover, only a few studies were conducted in full‐threshold clinical populations, leading to wide confidence intervals and high uncertainty in effect estimates. Accordingly, no disorder‐specific analyses could be conducted. Therefore, there is an urgent need for more research on the effectiveness of self‐help interventions in improving general mental health outcomes for clinically diagnosed populations. In this context, future research should balance the advantages of automated inclusion procedures based on screening questionnaires, known for their high scalability and easy accessibility, with studies that apply formal diagnoses, allowing for more precise conclusions regarding effects within specific populations and disorders.

Lastly, the meta‐analysis by Linardon, Jarman, et al. (2025) again accentuates our limited understanding of the mechanisms of change in the treatment of eating disorders. The current findings reveal significant variation among studies in terms of the included populations (e.g., inclusion criteria based on diagnoses vs. symptom levels with different cut‐offs), intervention contents (different lengths, concepts, and application methods), and outcomes (e.g., five different measures for depression). Despite these differences across studies, the estimated effect sizes by Linardon, Jarman, et al. (2025) exhibited relatively low heterogeneity (when considering the differentiation between pre‐selected and clinical samples). This suggests that variations in study and intervention characteristics did not result in markedly divergent effect sizes, indicating a consistent improvement in general mental health outcomes across different interventions. As the small number of studies limits the interpretability of the heterogeneity index and precludes subgroup or meta‐regressive analyses, these findings raise crucial questions about possible moderators and mediators. Consequently, the current evidence does not yet allow us to determine whether there are treatment‐specific mechanisms of change or a nonspecific treatment effect across all interventions. A limited understanding of the mechanisms of change may be particularly significant when investigating general mental health outcomes, as there are potentially different pathways of change (Linardon, Jarman, et al. 2025): Reducing symptoms of eating disorders may lead to improvements in overall mental health, or vice versa; alleviating general distress may decrease eating disorder pathology. Additionally, and most realistically, there may be interactive effects or shared underlying processes. In sum, understanding the mechanisms and directions of change is essential for comprehending the effects of self‐help interventions.

## 2 Future Directions: From Treatment Processes to Better Treatment Effects?

In sum, the meta‐analysis by Linardon, Jarman, et al. (2025) highlights that, on average, self‐help interventions can decrease the burden and comorbidities of eating disorders. This adds to other findings indicating their effectiveness in reducing eating disorder symptoms (Linardon, Liu, et al. 2025). However, the effects of self‐help interventions on general health outcomes were small, and we do not know how, why, and for whom they work. Moving forward, research on self‐help interventions might benefit from adapting ideas from modern developments in psychotherapy research to enhance their effectiveness in reducing general mental health symptoms and improving quality of life (Hofmann and Hayes 2019). This might enable a better transfer to clinical practice, helping treatment‐seeking patients and healthcare professionals with single‐case treatment decisions.

The applications of self‐help interventions range from specialized treatment for individuals diagnosed, potentially being alternatives to face‐to‐face therapy, to prevention‐focused interventions designed to reach as many participants as possible. Furthermore, self‐help interventions can vary significantly in (a) the form of delivery, from textbook to apps and potentially AI‐based methods; (b) the therapeutic content, which ranges from classical cognitive behavioral therapy to third‐wave or process‐based approaches; (c) the level of therapeutic contact, from no contact to blended care; and (d) the treatment focus, which can be symptom‐specific or transdiagnostic. Moreover, self‐help interventions facilitate the recruitment of larger and more representative samples. Still, so far, self‐help treatments for eating disorders have not been sufficiently tested across diverse populations. Most existing research has relied on samples composed predominantly of highly educated, young, white females, thereby neglecting individuals with different gender identities, cultural backgrounds, or socioecological contexts (Linardon, Jarman, et al. 2025). This sampling bias adds to the myth that eating disorders only affect a particular group of patients, increasing stigma and treatment barriers for underrepresented groups. However, by leveraging modern technologies enabling fast translations and international recruitments, web‐based interventions could be disseminated more widely, reaching more diverse samples.

Overall, advances in the field and an increasing number of studies might lead to even more variation across interventions, research designs, and populations. Consequently, self‐help interventions for eating disorders should not be considered a unified treatment approach; instead, their distinct differences should be acknowledged. As such, future studies would benefit from clarifying their objective(s) by outlining the intended role, primary, adjunctive, or preventive, of the tested intervention in the context of eating disorder treatment. On top of that, research designs should be well‐described and systematically tailored to this objective, including outcomes, population, sample size, inclusion criteria, and control groups. This may enable future meta‐analyses to conduct sophisticated subgroup or meta‐regressive analyses addressing the question of what works for whom.

Furthermore, future primary studies on self‐help interventions should also emphasize change processes. In traditional pre‐post research designs, mediator analyses are often limited in causal interpretation. However, these analyses could provide initial insights into change processes and the relationship between changes in different eating disorder–related outcomes. Moving forward, the flexibility and scalability of self‐help interventions may be particularly well‐suited for advancing research on mechanisms of change (Hofmann and Hayes 2019). Thus, future studies should utilize research designs that are better suited and more powerful for testing treatment effects and processes (Watkins and Newbold 2020). As data assessments can be smoothly integrated into technology‐based self‐interventions, they enable more frequent assessments, such as weekly questionnaires, leading to a higher temporal resolution than traditional pre‐, post‐, and follow‐up measurement designs. Hence, these designs may help identify smaller effects and more complex temporal relationships. Additionally, ecological momentary assessment designs can assess changes and underlying processes in real‐life conditions, reducing recall biases and enhancing ecological validity. Complementing these methods, component or factorial designs could be implemented to investigate the specific effects of certain therapeutic components and their interactions. As the content in self‐help interventions is highly standardized and divided into different modules, systematic variations in treatment presentation could be tested while controlling for covariates (Watkins and Newbold 2020). In sum, technological advances may enable the implementation of modern research designs, leading to more distinctive findings on treatment effects and their underlying processes.

Additionally, research on the effectiveness of self‐help interventions on general mental health may benefit from a transdiagnostic perspective. As already outlined by Fairburn over 20 years ago, targeting underlying dysfunctional mechanisms is essential for successfully treating eating disorders (Fairburn et al. 2003). However, although Fairburn's transdiagnostic model highlights the shared mechanisms among eating disorders, future research could benefit from generalizing the idea of underlying dysfunctional processes across more mental disorders. Eating disorders are often associated with comorbid disorders, and network analyses have shown that anxiety and depression are key symptoms within eating disorder networks. Thus, focusing on transdiagnostic mechanisms, a perspective also suggested by the Research Domain Criteria (RDoC), could generalize treatment effects and enhance overall well‐being. Promising transdiagnostic treatment targets may include cognitive, behavioral, or emotion‐regulation processes. Indeed, as self‐help interventions are modularized, they may be particularly well‐suited for transdiagnostic, flexible, personalized treatments, which could enhance their effectiveness in terms of quality of life and acceptability. To achieve this, intensive longitudinal data assessments can be utilized to obtain idiographic symptom and/or process networks, enabling the identification of individual treatment targets (Hofmann and Hayes 2019). Furthermore, treatments delivered via apps can facilitate the transfer to real‐life settings, as ecological momentary or just‐in‐time interventions can directly target dysfunctional processes in real time.

## 3 Conclusion

In conclusion, the findings by Linardon, Jarman, et al. (2025) underline the potential of self‐help interventions in treating eating disorders, as they can effectively reduce eating disorder symptoms and potentially target comorbid symptoms, general distress, and well‐being. Thus, self‐help interventions may enhance routine care and reduce the global eating disorder burden. Building upon these promising findings, the aim of future research on self‐help treatments for eating disorders should be to develop a more comprehensive understanding of treatment processes. By identifying and targeting transdiagnostic mechanisms of change, we may be able to increase the overall clinical utility of self‐help interventions for eating disorders and enhance patients' well‐being. Adopting ideas from other (psychotherapy) research fields might contribute to more accessible, effective, and personalized treatment options. Promisingly, many of the outlined ideas have already been implemented in eating disorder research, leading to the optimistic conclusion that advances in psychotherapy research might further contribute to step‐by‐step closing the treatment gap for eating disorders.

## Author Contributions

**Steffen Hartmann:** conceptualization, writing – original draft, writing – review and editing.

## Conflicts of Interest

The author declares no conflicts of interest.

## Linked Articles

This article is linked to Jake Linardon et al. papers. To view this article, visit https://doi.org/10.1002/eat.24405.

## Data Availability

Data sharing not applicable to this article as no datasets were generated or analysed during the current study.
